# Word sense disambiguation of acronyms in clinical narratives

**DOI:** 10.3389/fdgth.2024.1282043

**Published:** 2024-02-28

**Authors:** Daphné Chopard, Padraig Corcoran, Irena Spasić

**Affiliations:** School of Computer Science and Informatics, Cardiff University, Cardiff, United Kingdom

**Keywords:** natural language processing, word sense disambiguation, acronym disambiguation, machine learning, deep learning, silver standard

## Abstract

Clinical narratives commonly use acronyms without explicitly defining their long forms. This makes it difficult to automatically interpret their sense as acronyms tend to be highly ambiguous. Supervised learning approaches to their disambiguation in the clinical domain are hindered by issues associated with patient privacy and manual annotation, which limit the size and diversity of training data. In this study, we demonstrate how scientific abstracts can be utilised to overcome these issues by creating a large automatically annotated dataset of artificially simulated global acronyms. A neural network trained on such a dataset achieved the F1-score of 95% on disambiguation of acronym mentions in scientific abstracts. This network was integrated with multi-word term recognition to extract a sense inventory of acronyms from a corpus of clinical narratives on the fly. Acronym sense extraction achieved the F1-score of 74% on a corpus of radiology reports. In clinical practice, the suggested approach can be used to facilitate development of institution-specific inventories.

## 1 Introduction

Acronyms are formed as systematic abbreviations of frequently mentioned words and phrases, which follow special capitalisation and blending patterns (1). Their primary purpose is to make written communication more efficient in terms of space and time. The readers’ familiarity with specific acronyms rather than their orthographic regularity plays a critical role in rapidly interpreting their intended meaning (2). Therefore, not only do the globally accepted acronyms pose no major difficulties in specialist communication, but they in fact facilitate such communication between domain experts. For example, clinical narratives feature extensive use of acronyms, which are not defined explicitly in the corresponding documents.

Even though such acronyms pose no difficulty to their intended users, they can still hinder the performance of natural language processing (NLP) algorithms (3, 4) when clinical narratives need to be analysed automatically. For example, the use of acronyms (e.g. “DVT”) obscures the corresponding phrase (e.g. “deep vein thrombosis”) whose constituents (e.g. “thrombosis”) cannot be indexed and, hence, cannot be retrieved. On the other hand, clinical acronyms are highly polysemous (5), e.g. “ED” can be interpreted as “eating disorder,” “elbow disarticulation,” “emotional disorder,” “emergency department” or “erectile dysfunction,” which may cause irrelevant documents to be retrieved when using the acronym as a search term. These problems can be resolved by automatically mapping acronyms to their correct senses in an external dictionary (6) based on their context of use.

This may be viewed as a word sense disambiguation (WSD) problem (7), commonly approached by supervised learning approaches, which are trained using a set of annotated examples. Their performance depends largely on the amount of annotated data used for training. However, the data annotation bottleneck presents one of the key obstacles to supervised learning approaches in clinical NLP (8). Patient privacy concerns further narrow down this bottleneck by removing the possibility of crowdsourcing annotation. Crowdsourcing remains an option for annotating synthetic data, but it is difficult to scale when medical expertise is required for accurate annotation.

To eliminate the manual data annotation bottleneck altogether, we looked at the possibility of generating an annotated dataset automatically. Namely, scientific writing conventions prescribe that all acronyms need to be explicitly defined the first time they are mentioned. Local acronym definitions can be removed from text and used instead as sense labels. By doing so, we can simulate the clinical narrative style of acronym usage and create a large dataset that can be used to train supervised approaches to WSD of clinical acronyms. This leads us to the main contribution of this study. We describe an algorithm for WSD of clinical acronyms, which offers two key novelties. First, it requires no manual annotation of data, be them clinical or otherwise. Second, it requires no predefined sense inventory and instead uses the corpus itself to extract potential senses on the fly.

## 2 Related work

Acronym-related NLP tasks can be divided into three groups (9). Acronym definition identication is concerned with finding pairs of acronyms and their long forms that are defined in the input text. Similarly to named entity recognition, acronym identification takes a text as input and tags the spans of acronyms and long forms using the BIO format (short for beginning, inside, outside). Finally, acronym disambiguation takes a sentence that contains an ambiguous acronym as input and aims to select an appropriate long form from a predefined set of candidates. The first task was proposed long before the other two and has attracted a lot of attention, especially in the biomedical domain, where a variety of rule-based algorithms have been developed with great success (10, 11).

Acronym identification and acronym disambiguation tasks were introduced recently at the AAAI Workshop on Scientific Document Understanding (12). Given the success of neural networks with a transformer architecture in a variety of NLP tasks, it was not surprising that the majority of participants opted for such an approach, which they used to model acronym disambiguation as binary classification (13, 14), information retrieval (15) or span prediction (16). They provided the best performance with F1-scores ranging between 91.58% and 94.05% lagging slightly behind the human performance of 96.10%. Other types of neural networks architectures provided similar performance of 91.34% (17). Traditional machine learning approaches performed at 88.40% at best, struggling mostly in terms of recall (18, 19).

Clinical domain, in which almost one third of abbreviations were found to be ambiguous (20), is very much in need of effective acronym disambiguation methods. Approaches such as the ones described above depend on predefined sense inventories. Unfortunately, the two largest sense inventories for biomedical acronyms, the Specialist Lexicon Release of Abbreviations and Acronyms (LRABR) (21) and Another Database of Abbreviations in MEDLINE (ADAM) (22), were shown to have micro-coverage of acronyms in clinical narratives of 74.8% and 68.0%, respectively. Micro-coverage of long forms was found to be 82.5% and 55.4%, respectively. Disambiguation cannot be performed when an appropriate sense is missing from the inventory. To address this issue, a method was developed to automatically harmonise sense inventories from different regions and specialties into a comprehensive inventory (23), which improved micro-coverage of acronyms and their senses to 94.3% and 99.6%, respectively.

Early approaches to disambiguation of clinical abbreviations and acronyms, which were based on rules (24) and traditional machine learning (25–28), performed with moderate accuracy ranging between 50.1% and 75% (29). Despite the obvious improvements that deep neural networks offer to acronym disambiguation (12), there is evidence that rule-based approaches may not only outperform them but error analysis even suggests why (9). Namely, end-to-end machine learning commonly emphasise certain types over others and by doing so fail to take advantage of orthography, which plays an important role in the formation of acronyms. Deep neural networks can capture orthographic constraints by integrating token-based and character-based networks.

## 3 Material and methods

### 3.1 System overview

**Figure 1:**
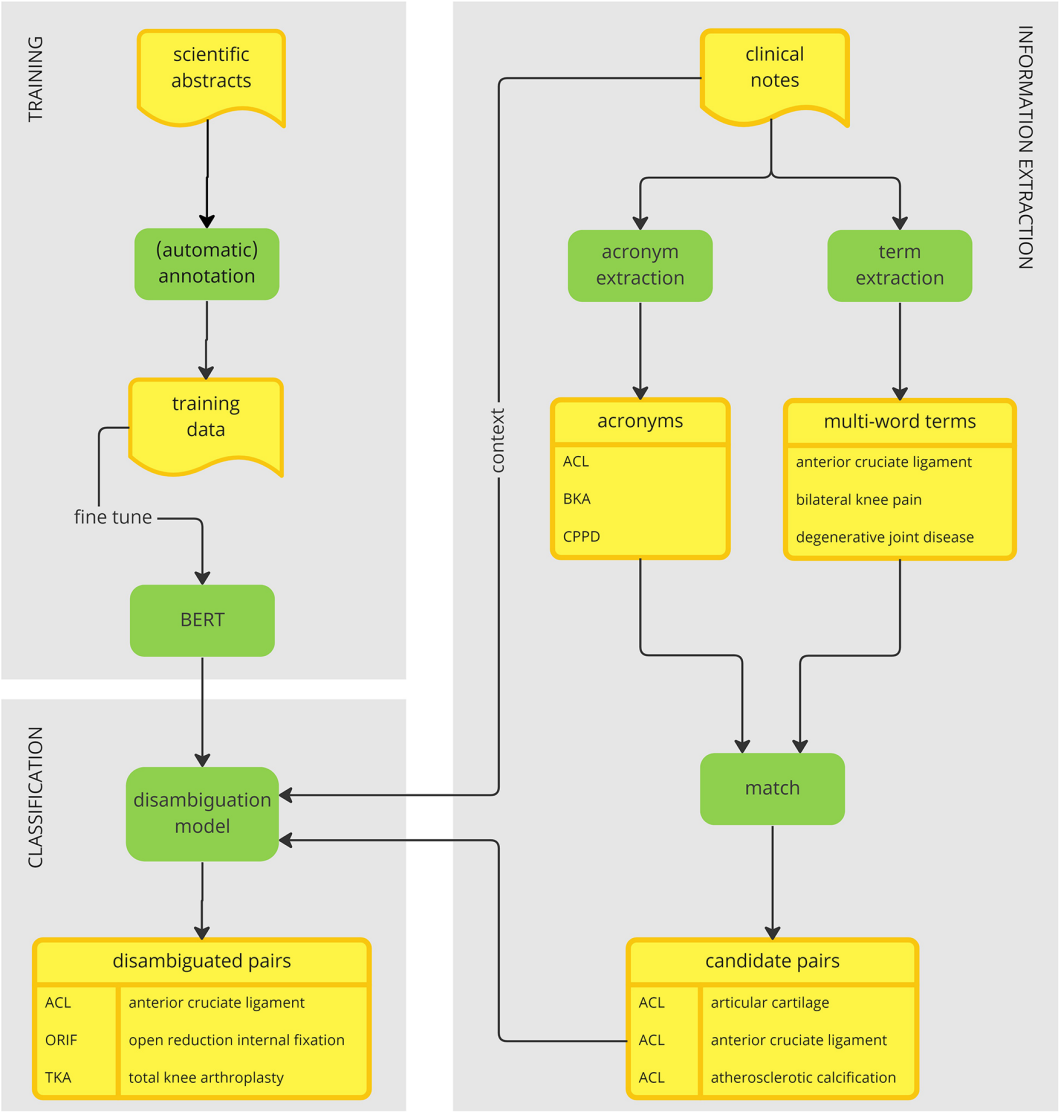
An acronym disambiguation framework.

The overall framework of our acronym disambiguation approach is provided in Figure 1. The system has two main modules, one treating WSD as a binary classification problem (shown on the left-hand side) and the other one dedicated to extracting acronyms and their potential senses from text (shown on the right-hand side). Given a pair consisting of an acronym within its context and a potential long form, binary classification is performed to determine whether the long form represents a correct interpretation of the acronym or not. In this framework, the binary classification module is used to resolve the ambiguity of acronyms arising from extracting multiple long form candidates from the corpus.

The binary classifier is trained on a large corpus of scientific abstracts. This choice of data serves two main purposes. First, it bypasses the privacy concerns associated with clinical data. Second, the scientific writing conventions allow for an easy simulation of global acronyms, whose sense can be annotated automatically. These two factors combined together allow for the creation of a large annotated dataset that can be used to train a deep learning model for disambiguation of global acronyms. To train the model, we fine-tuned Bidirectional Encoder Representations from Transformers (BERT) (30), a large pretrained language model, to perform binary classification as indicated above.

To link acronyms to their long forms found within clinical narratives, we start by extracting potential acronyms on one side and extracting potential long forms on the other side. These two activities are independent of each other and hence can be performed in parallel. We extract potential acronyms using a simple heuristic based on their orthographic properties. We extract multi-word terms (MWTs) from text based on their linguistic and statistical properties using a method called FlexiTerm (31). Having retrieved both acronyms and MWTs, they are matched using their internal properties. In a nutshell, the characters from an acronym are aligned against each MWT to shortlist potential long forms.

All candidate pairs of acronyms and their potential long forms are then disambiguated using the previously trained disambiguation model based on the context in which the acronym is mentioned. The final result is an inventory of acronyms mapped to their senses. The inventory is derived directly from the corpus. In that aspect, our method departs from the traditional acronym disambiguation approaches, which rely on an external inventory to obtain a list of possible senses.

The following sections provide further details about each module of the proposed framework.

### 3.2 Binary classification of acronym senses

#### 3.2.1 Data collection and annotation

Data collection and annotation were performed using a web-based application for simulation and annotation of global acronyms (32), whose long forms are not defined explicitly in text. This application automatically modifies PubMed abstracts to simulate global acronym usage by removing their explicitly defined long forms and using them to annotate their senses without any need for external lexicons or manual annotation. Thus, it can be used to create large training datasets for WSD of biomedical acronyms. Given the automated extraction of acronym definitions, such data will inevitably contain some degree of noise, which based on the performance of the underlying algorithm (10) is estimated to be around 4%.

We started by defining a PubMed search query that targeted the journals that cover clinical applications. We combined the keyword “clinical” with the suffix “-logy” to refer to various clinical domains (e.g. “rheumatology”) while also explicitly excluding certain keywords (e.g. “biology”). The corresponding abstracts were downloaded from PubMed and annotated automatically. We downloaded the sense inventory, which was also generated automatically by the web-based application. Each acronym associated with a single long form in the sense inventory was discarded as it was unambiguous and thus irrelevant for the disambiguation task at hand. A total of 963 unique acronyms remained, each having between 2 and 27 possible long forms with the mean of 4.09 and the median of 3 long forms.

Given an acronym’s potential long form, we choose to tackle WSD as a binary classification task. To properly train such a WSD model, we needed both positive examples, where the long form candidate is correct, and negative examples, where the long form candidate is incorrect. Using the previously annotated acronyms, we proceeded as follows. First, we extracted every sentence containing an annotated acronym. For each sentence, we created two examples: a positive example corresponding to the original triplet (sentence, acronym, correct long form) and a negative example, in which the correct long form was replaced at random by another long form according to the dictionary created in the previous step. In this manner, we obtained a balanced dataset of positive and negative examples, both in the form of a triplet (sentence, acronym, long form).

Prior to training a WSD model, we set aside a portion of the data for validation, i.e. to evaluate the model’s fit throughout its development including hyperparameter optimisation. For each of the 963 ambiguous acronyms, a total of 1,000 examples (or 10% if fewer than 10,000 examples were available) were randomly selected and reserved for validation. We repeated this process on the remainder of the data to obtain a test set. A total of 16,130,782 remaining examples were used for training.

#### 3.2.2 Training a disambiguation model

**Figure 2:**
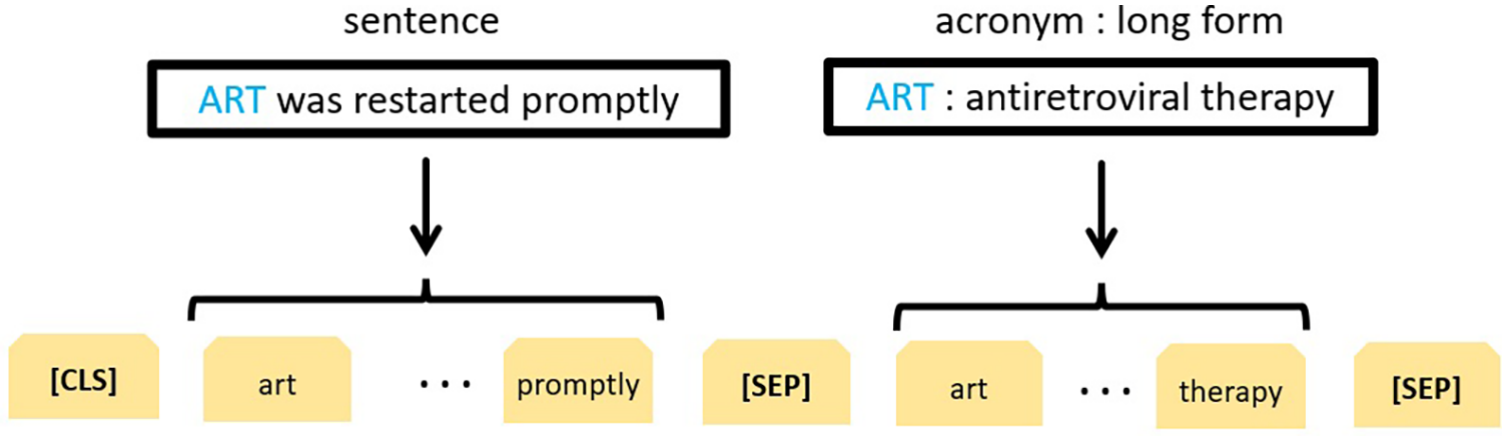
BERT-based representation of the acronym disambiguation problem.

Recall that each example is in the form of a triplet (sentence, acronym, long form). The long form needs to be classified to determine whether or not it represents a correct interpretation of the acronym within the given sentence. For this task we used a transformer-based architecture similar to that of (33). This architecture fine-tunes BERT (30) to perform WSD given a sentence and one of its ambiguous words. More specifically, the model is fed two text sequences: (i) an ambiguous word within its context and (ii) that word followed by its gloss (one of many) from WordNet (34). In our case, the word and its gloss correspond to an acronym and its potential long form, respectively as shown in Figure 2. The rest of the BERT-based neural network architecture is identical to the one we previously used for binary classification of potential adverse events (35), where practical implementation details can be found. In summary, three types of embeddings were used: token embeddings to represent tokens, segment embeddings to differentiate between different segments in the input (see Figure 2) and position embeddings to encode the word order. For each token, these embeddings were added and passed onto to the pretrained BERT–BASE model comprised of 12 layers of transformer encoders, each having a hidden size of 768 and 12 attention heads. A token-specific output produced at each layer can be interpreted as its contextualised embedding. The final output produced for the initial [CLS] token, commonly used as an aggregate problem representation, was forwarded to the classification layer, but only after a 0.1 dropout to reduce overfitting. The two logits outputted by the classification layer correspond to the question of whether the given longform is a correct interpretation of the given acronym given its context or not. The weights of the classification layer were learned during BERT fine-tuning. They were initialized using a truncated normal distribution with mean 0 and standard deviation of 0.02. Next, a softmax function was applied to obtain the probability distribution of the two classes. The loss function (softmax cross entropy between the logits and the class labels) was optimized using the Adam optimizer with an initial learning rate of $2\times 10^{-5}$. The binary classification model was trained for 8 epochs. All other parameter values were identical to those used in the original BERT_BASE_ uncased model, including the clip norm of 1.0 and 100 warmup steps with linear decay of learning rate.

Of note, a similar approach developed by (13) was the winning solution for the second SDU@AAAI-21 shared task on acronym disambiguation in English scientific papers (12). They used a different problem representation with the long form as the first text sequence and the acronym within its context as the second sequence. To direct the model’s attention to the ambiguous acronym, they marked up the acronym using two special tokens (<start> and <end>).

### 3.3 Acronym sense extraction

The purpose of this section is twofold. First, it aims to demonstrate the effectiveness of the acronym disambiguation module trained on a corpus of biomedical abstracts using a corpus of clinical narratives. Second, it extends the functionality of acronym disambiguation to include the extraction of potential acronyms and the corresponding long form candidates automatically from a given corpus.

#### 3.3.1 Data collection and annotation

To assemble a corpus of clinical narratives, we used MIMIC-III. This large, freely available database comprises de-identified health-related data associated with over forty thousand patients who stayed in critical care units of the Beth Israel Deaconess Medical Center between 2001 and 2012 (36). We retrieved a total of 2,609 knee radiology reports. This choice of clinical narratives was motivated by the availability of local expertise needed to interpret the results. Specifically, we had access to the team who developed the TRAK ontology (37), which defines standard care for the rehabilitation of knee conditions, and who previously applied this ontology to support text mining of knee radiology reports (38).

The raw reports were processed as follows. We extracted the main body of each report. A set of simple regular expressions were used to recognise section headings and remove them from further consideration. The main reason for discarding section headings was the subsequent recognition of potential acronyms. Section headings are usually written in uppercase, which is one of the properties used to identify acronyms.

**Figure 3:**
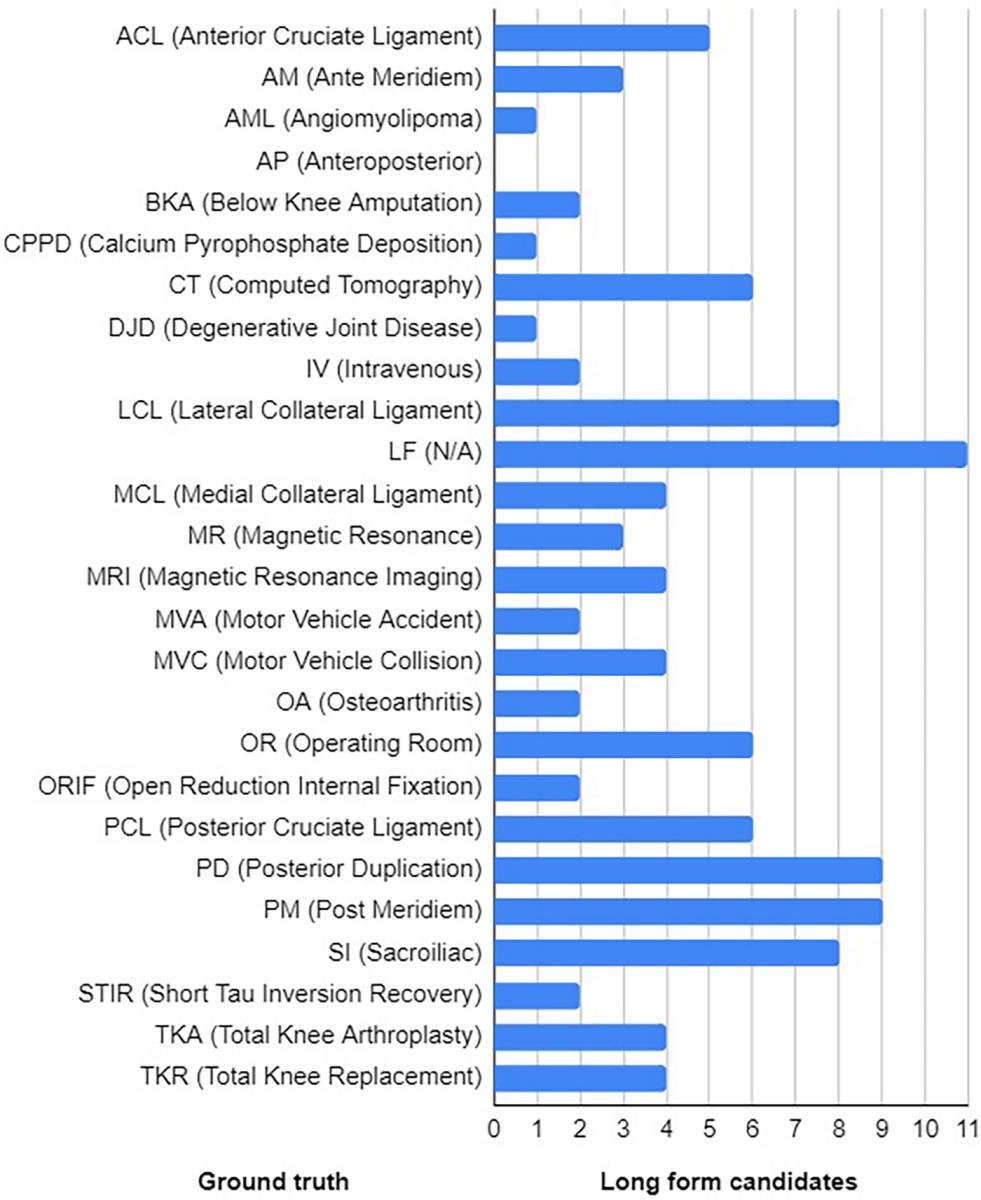
Ground truth with the distribution of long form candidates.

To recognise potential acronyms, we used a simple heuristic based on the rules described in Table 1, which, when tested on an inventory of 36,162 acronym definitions derived from (39), was able to match a total of 32,940 (91.09%) acronyms. Using this heuristic, we extracted a total of 26 potential acronyms from the corpus, which were then interpreted manually by analysing their concordances (see Figure 3). One of the potential acronyms, LF, turned out to be a special token used in MIMIC-III to indicate missing information that had been removed to anonymise the data. Hence its long form is not available in the ground truth. Nonetheless, we retained it as a potential acronym to challenge the disambiguation model to reject potential long forms as false positives. Further, the long forms of three acronyms, IV, OA and SI, consist of a single word. In this study, we are exploring only MWTs as potential long forms. Thus, when these acronyms are paired with MWTs as potential long forms, we know in advance that it can result in either a false positive or a true negative.

**Table 1:** Heuristic rules for acronym recognition.

ID	Rule	Rationale
1	The token has to be 2–10 characters long.	Acronyms are short forms.
2	The token has to be tagged as a noun.	Acronyms are matched against MWTs, which are noun phrases.
3	The first character of the token has to be a capital letter.	Apart from few exceptions (e.g. “mRNA”) most acronyms start with a capital letter.
4	The number of letters has to be greater than the number of digits.	To prevent retrieving numerical expressions (e.g. “USD5000”).
5	The number of uppercase letters has to be greater than the number of lowercase letters.	To prevent retrieval of outliers such as personal names (e.g. “McMurray”) and titles (e.g. “Miss”).
6	The token has to occur at least 10 times in the corpus.	Acronyms are introduced because the corresponding concepts are referred to frequently.

To find potential long forms within the corpus, we applied a method called FlexiTerm, which was originally developed to recognise MWTs (31). Even though it was subsequently extended to recognise acronyms as MWTs (40), we did not use this option in order to test acronym disambiguation with our newly developed model. The list of MWTs was obtained using the latest implementation of FlexiTerm (41). It consisted of 2,079 terms, each linked to multiple variants arising from inflection, derivation or hyphenation.

To match potential acronyms and MWTs, we used a simple heuristic based on the rules described in Table 2. When tested on an inventory of 32,940 definitions whose acronyms matched the rules described in Table 1 (see above), a total of 31,241 (94.84%) acronym definitions were consistent with the patterns described in Table 2. Of course, all ambiguous acronyms will match most of the corresponding long forms and potentially other phrases extracted from the corpus. At this stage, the matching procedure was deliberately loose so as to extract as many potential long forms in order to challenge the disambiguation model. As a result, we assembled a list of 110 pairs of acronyms and their potential long forms. Each acronym was mapped to 4.19 MWTs on average with standard deviation of 2.91 (see Figure 3 for distribution).

**Table 2:** Heuristic rules for matching acronyms and MWTs.

ID	Rule	Rationale
1	Both acronym and MWT have to start with the same letter.	Apart from few exceptions (e.g. “XML”), most acronyms start with the initial letter of the long form.
2	All characters from the acronym have to occur in the same order within the MWT.	Acronyms are formed by removing characters from the long form, which preserves their original order.
3	The acronym cannot occur as a token within the long form.	Acronym cannot abbreviate itself.
4	The last character from the acronym has to occur in the last token of the MWT.	With the exception of prepositions, which cannot occur at the end of a noun phrase, most other tokens are referenced by a character within the acronym.
5	The Damerau–Levenshtein distance between the acronym and the initialism constructed out of the MWT is less than 2.	To ensure that most initial characters correspond to a character within the acronym.

Having paired acronyms and long forms, each pair was then labelled as either positive or negative example according to the ground truth provided in Figure 3. All terms that conveyed the sense of the long form provided in the ground truth were annotated as positive examples. For example, terms “total knee arthroplasty” and “total knee replacement” are synonyms and as such they convey the same sense as the acronym “TKA.” Similarly, “total left knee arthroplasty” is a “total knee arthroplasty” and as such is accepted as a correct sense of “TKA.” The ground truth was extended by manually annotating all 110 pairs of acronyms and their potential long forms (see Supplementary Material).

Recall that the disambiguation model expects a triplet (sentence, acronym, long form) as its input (see Figure 2). Having paired acronyms and long forms, for each acronym we extracted all sentences that mention the acronym. Given that the corpus of clinical narratives belonged to a single domain of knee radiology reports, we used one sense per discourse hypothesis (42) to propagate the label originally assigned to the pair (acronym, long form) to all corresponding triplets (sentence, acronym, long form).

### 3.4 Binary classification of acronym senses

All triplets (sentence, acronym, long form) extracted from clinical narratives were passed through the BERT-based disambiguation model trained on scientific abstracts described in Section 3.2.2. For each (acronym, long form) pair, individual classifications of acronyms occurrences in the corresponding sentences were aggregated. A long form was deemed positive if it was classified as such in at least 75% of the acronym occurrences; otherwise it was deemed negative. In this manner, a sense inventory is constructed (see bottom left in Figure 1).

## 4 Results

### 4.1 Binary classification of acronym senses

Collocational stability refers to the consistency and predictability of words that tend to appear together. High frequency of multi-word phrases implies their collocational stability (43), which in turn enhances semantic clarity of a discourse. Acronym definitions themselves are collocations (44). Given that, in our approach, potential long forms are not sourced from a predefined inventory but are instead extracted from a corpus using a method that measures their collocational stability (31, 41), it is intuitively plausible to assume that the most frequent one is indeed the correct one. In other words, whenever multiple MWTs extracted by FlexiTerm match a given acronym according to the rules described in Table 2, the baseline method selects the most frequent one.

We evaluated the BERT-based disambiguation model described in Section 3.2.2 on the test subset of PubMed abstracts. The results provided in Table 3 were compared against a baseline classifier described above. At almost 95% across all metrics, our model outperformed the frequency-based approach indicating that it successfully identified patterns for acronym disambiguation. While this disambiguation model proved to be both effective and efficient, its main drawback comes from the supervised training as it may struggle to disambiguate acronyms that were not represented in the training data. This is a valid concern when a model trained on scientific abstracts is applied on genuine clinical data. The next section evaluates the performance of acronym sense extraction, which incorporates binary classification, on clinical data, hence offering an insight into its performance of such data.

**Table 3:** Performance of the acronym disambiguation model on scientific abstracts.

Method	Accuracy	Precision	Recall	F1-score
Baseline	64.96%	69.15%	54.02%	60.65%
BERT	94.62%	94.45%	94.89%	94.67%

### 4.2 Acronym sense extraction

The first row of Table 4 provides the results of acronym sense extraction on clinical narratives. Before interpreting the evaluation results, it is worth pointing out that one major difference between the two evaluations. The evaluation results on the set of scientific abstracts shown in Table 3 were obtained on a balanced set where the number of positive and negative test examples was equal. The balance was achieved by design. When evaluating acronym sense extraction, the distribution of potential senses is a function of the corpus from which these senses were extracted. Not surprisingly, the distribution of correct and incorrect senses was heavily skewed in favour of the incorrect ones, which outnumbered the correct senses by a ratio of over 5:1. In an attempt to redress the extreme imbalance, we tried to filter the long forms prior to passing them on to the binary classifier for disambiguation. For each acronym, we kept only the top two matching MWTs according to their termhood score calculated by FlexiTerm under an assumption that acronyms are more likely to be introduced for collocationally stable phrases. This somewhat reduced the number of incorrect senses, which, however, still outnumbered the correct senses by a ratio of over 3:1. The corresponding evaluation results are provided in the second row of Table 4.

**Table 4:** Performance of the acronym disambiguation model on clinical narratives.

Long forms	Accuracy	Precision	Recall	F1-score
All terms	84.40%	55.26%	100.00%	71.19%
Top 2 terms	80.85%	59.09%	100.00%	74.29%

Accuracy is a poor measure for imbalanced data as any model that predicts the majority class for all predictions is bound to achieve a high classification accuracy. We, therefore, turn our attention to precision and recall. We can immediately observe the perfect recall, which indicates that none of the correct senses were misclassified. However, the precision of at most 60% indicates a high number of false positives. Error analysis revealed an interesting fact. The majority of false positives shared a word (or a subword) with the corresponding true positive. Consider for example, “lateral condyle” and “medial condyle,” which were incorrectly classified as long forms of the acronyms MCL (medial collateral ligament) and LCL (lateral collateral ligament). In both cases, the false and true positives share the first word. A similar pattern can even be observed at the subword level. For example, “intraoperative views” was incorrectly classified as a long form of IV (intravenous). Similarly, “osseous abnormality” was incorrectly classified as OA (osteoarthritis). “Osseous” is an adjective meaning “bony,” whereas “osteo-” is a combining form meaning “bone.”

The masked language modelling, which is used to pretrain BERT, may provide a plausible explanation for this phenomenon. During pretraining, 15% of the words are masked randomly forcing the model to predict the output for those words based on the words around them. When BERT encounters an acronym, especially if it has not been seen during pretraining, it will try to predict its embedding based on its context. As the long form of an acronym is fully compatible with the context of the acronym, when some of the corresponding words (or subwords) are found in a potential long form, the classification model may use them as evidence for positive classification.

Finally, let us address our previous concern of applying a disambiguation model trained on scientific abstracts on genuine clinical data. Out of 26 acronyms extracted from the test data (clinical narratives), nine were not found in the training data (scientific abstracts). Despite this, the classifier correctly disambiguated all but two of them. In line with our previous observation about the masked language model, this suggests that the model was able to exploit the words in the long form to disambiguate an acronym based on its context.

## 5 Discussion

We described a system for WSD of global acronyms in clinical narratives. It integrates a rule-based method for data annotation, a statistical method for MWT recognition, a rule-based method for extracting a sense inventory from a corpus and, finally, a neural network for disambiguation. These methods complement one another in a way that offers multiple advantages over the state of the art. First, it uses scientific abstracts to simulate clinical narrative style of acronym usage and annotate them automatically with the correct senses. This circumvents the problems associated with patient privacy and manual annotation overhead, which have traditionally plagued the use of clinical text data in machine learning (8). In turn, large amounts of annotated data lay a foundation for training a robust neural network, which fosters trust and reliability necessary for real-world clinical applications. Another practical advantage of our approach is that it does not require predefined sense inventories, which are known to vary greatly across different geographic regions and medical specialties (23). Instead, an inventory of potential senses is extracted from a corpus on the fly. This does not only make our approach readily portable across different domains, but also improves its utility in clinical practice. For example, our approach can be used to facilitate development of institution-specific inventories, which in turn can be used to improve communication and shared decision making.

CLASSE GATOR (CLinical Acronym SenSE disambiGuATOR) (45) is the most similar system for WSD of clinical acronyms. The key similarity is in the use of scientific literature for bootstrapping training data. The key difference is that their clinical acronym senses are also extracted from the literature. This fact requires the two corpora of scientific literature and clinical narratives respectively to belong to the same domain. It also requires the acronym sense to be used in the literature. The main criticism of previous acronym disambiguation systems is that they constructed a sense inventory from a single institutional corpus, which hindered their generalisability, because acronyms tend to vary across institutions (23). Therefore, scientific literature cannot reasonably be expected to cover acronyms as they are used in clinical practice. Nonetheless, the two systems advance the area of acronym disambiguation in complementary ways.

Both CLASSE GATOR and our own approach are based on BERT, one of many large pretrained language models. It was fine-tuned for the WSD task using a scientific corpus as a proxy for clinical narratives, which remain largely inaccessible due to privacy concerns. However, recent advances in generative language models have opened up a new opportunity for training NLP models in a clinical domain. What sets apart generative models from other types of language models is their ability to produce original content. This can be used to generate potentially unlimited amounts of training data. Early efforts have demonstrated that even though the generated text was of poorer quality relative to the original text, when it was used to augment the training data, it still boosted the performance of downstream NLP tasks (46). Domain-specific databases can be used to mitigate the inherently stochastic nature of large language models (47) in an attempt to improve the accuracy, diversity and complexity of generated clinical data (48). Nonetheless, the data annotation bottleneck still persists but may be addressed with strategic prompt engineering. Our future work will explore this opportunity to make further advances in WSD of clinical acronyms.

While our evaluation demonstrates the generalisability of our approach in that it can successfully disambiguate acronyms that have not been seen in the training data, it certainly stands to further improve its performance by utilising the context of long form candidates. Further performance improvements may be gained by modeling the orthographic constraints of acronym formation. This would help eliminate some of the false positives. Therefore, we will be looking to extend the binary classification component by integrating token-based and character-based neural networks.

## Data Availability

The raw data used in this study can be downloaded from PubMed (https://www.nlm.nih.gov/databases/download/pubmed_medline.html) and MIMIC-III (https://physionet.org/content/mimiciii/1.4/). The data can be processed using open-source tools, which can be downloaded from GitHub (https://github.com/ispasic/acronimity; https://github.com/ispasic/FlexiTerm-Python).
